# Case Report: Heparin-induced thrombocytopenia leads to acute myocardial infarction post-PCI in multi-vessel coronary artery disease

**DOI:** 10.3389/fcvm.2026.1650624

**Published:** 2026-02-02

**Authors:** Zishan Wang, Xingpo Li, Xue Xu, Bin Yi, Hongxia Yu

**Affiliations:** Cardiovascular Medicine Department, Huantai County Hospital of Traditional Chinese Medicine, Zibo, China

**Keywords:** acute myocardial infarction, case report, coronary artery disease, heparin-induced thrombocytopenia, PCI

## Abstract

Heparin-induced thrombocytopenia (HIT) is a rare but severe complication of heparin therapy, characterized by a significant reduction in platelet count and a paradoxical prothrombotic state, which increases the risks of both arterial and venous thrombosis. This case report describes a 58-year-old male patient with multi-vessel coronary artery disease who developed acute ST-segment elevation myocardial infarction (STEMI) following successful percutaneous coronary intervention (PCI). Despite initial successful revascularization, the patient experienced recurrent chest pain, and HIT was clinically suspected based on a significant drop in platelet count and the 4Ts scoring system, though confirmatory anti-PF4/heparin antibody testing was unavailable at our institution. Treatment with corticosteroids was initiated; however, following transfer to another hospital, the patient received platelet transfusion—a contraindicated intervention in HIT—and subsequently succumbed to a fatal arrhythmic event. This case highlights the diagnostic and therapeutic challenges of suspected HIT in PCI patients, where it may mimic other post-procedural complications such as stent thrombosis. It underscores the critical need for vigilant monitoring of platelet counts, timely access to confirmatory diagnostic testing, immediate initiation of guideline-recommended non-heparin anticoagulants, and seamless communication during inter-hospital transfers to prevent potentially harmful interventions and improve patient outcomes.

## Introduction

Heparin-induced thrombocytopenia (HIT) is a rare but potentially life-threatening immune-mediated complication, which was reported to occur in approximately 0.5%–5% of patients receiving heparin (1). It is characterized by a significant reduction in platelet count and a paradoxical prothrombotic state. This condition arises from the formation of antibodies against complexes of platelet factor 4 (PF4) and heparin, which leads to platelet activation and an increased risk of both arterial and venous thrombosis (2). In patients undergoing percutaneous coronary intervention (PCI), where heparin is commonly utilized for anticoagulation, HIT poses a unique diagnostic challenge due to its clinical presentation, which can mimic other post-procedural complications, such as stent thrombosis. The complexity of managing HIT in the context of PCI lies in balancing the need for anticoagulation to prevent stent-related complications against the risks of exacerbating thrombocytopenia or thrombosis. This case report discusses a 58-year-old male with multi-vessel coronary artery disease who developed HIT following successful PCI, resulting in fatal complications. Through this case, we aim to highlight the diagnostic and therapeutic challenges of HIT in the post-PCI setting and underscore the importance of vigilant monitoring and timely intervention to improve patient outcomes.

## Case presentation

**Figure 1:**
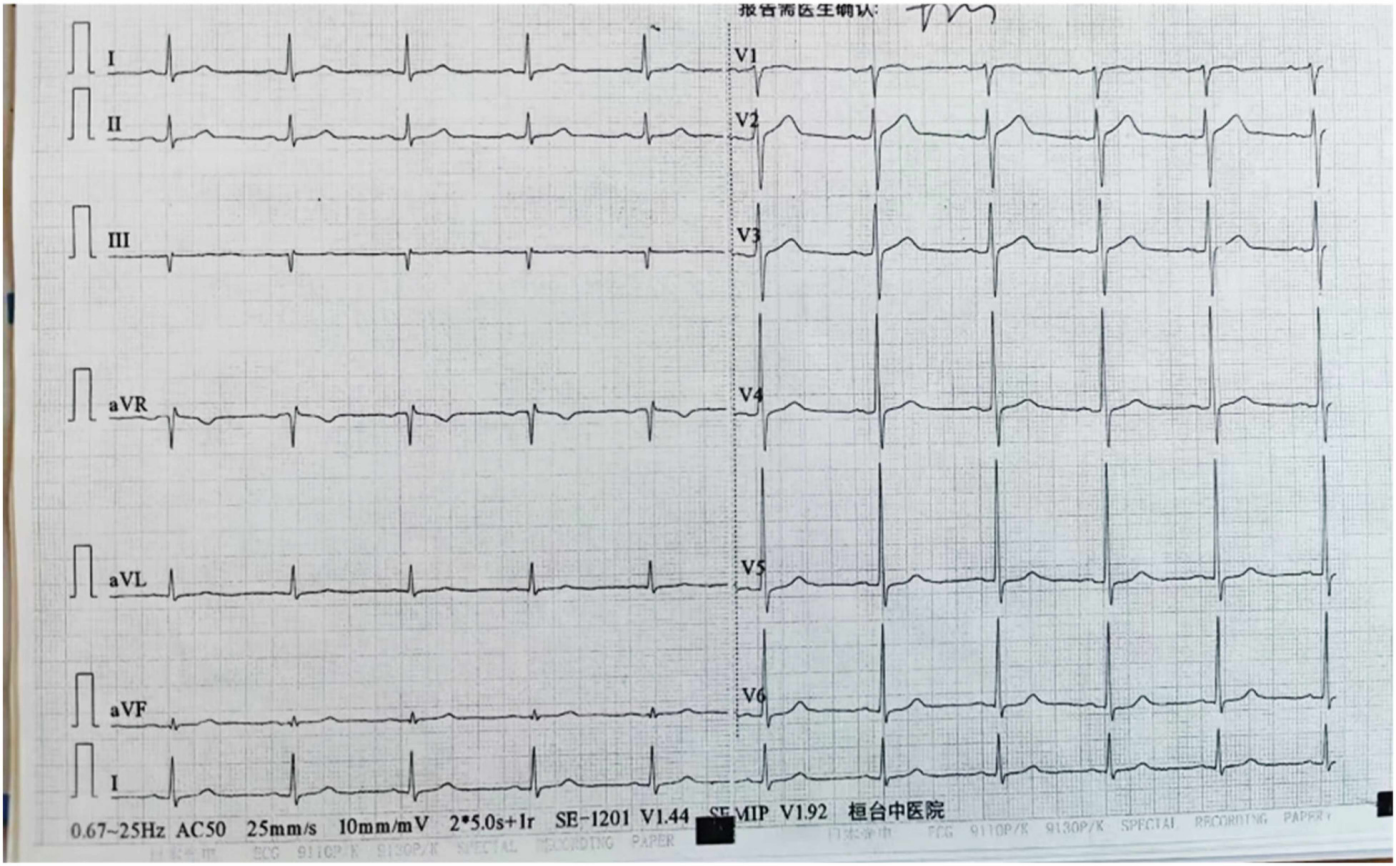
Electrocardiography (ECG) performed upon admission showed no significant abnormalities.

A 58-year-old male was admitted to the cardiology department of our hospital with a primary complaint of chest tightness and discomfort persisting for over one month. Approximately one month prior to admission, the patient began experiencing intermittent episodes of chest tightness without any identifiable triggers. These episodes were accompanied by radiating pain in the neck and were aggravated by physical exertion. Each episode typically lasted approximately10 min and resolved spontaneously. The patient had a notable medical history of essential hypertension for eight years, managed with felodipine 5 mg daily and aspirin 100 mg daily. Additionally, he had a long-standing history of hyperlipidemia, managed with atorvastatin calcium 20 mg at night. Electrocardiography (ECG) performed upon admission showed no significant abnormalities (Figure 1). Upon clinical evaluation and initial diagnostic tests, the patient was diagnosed with coronary atherosclerotic heart disease with unstable angina, essential hypertension, and hyperlipidemia. Physical examination on admission revealed no remarkable abnormalities. Laboratory tests showed the following levels: cardiac troponin I (0.02 ng/mL), myoglobin (76 ng/mL), NT-proBNP (126 pg/mL), creatine kinase (CK, 76 U/L), creatine kinase-MB (CK-MB, 15 U/L), total cholesterol (TC, 4.27 mmol/L), LDL-cholesterol (LDL-C, 2.63 mmol/L), fasting blood glucose (5.18 mmol/L), and platelet count (345 × 10^9^/L).

**Figure 2:**
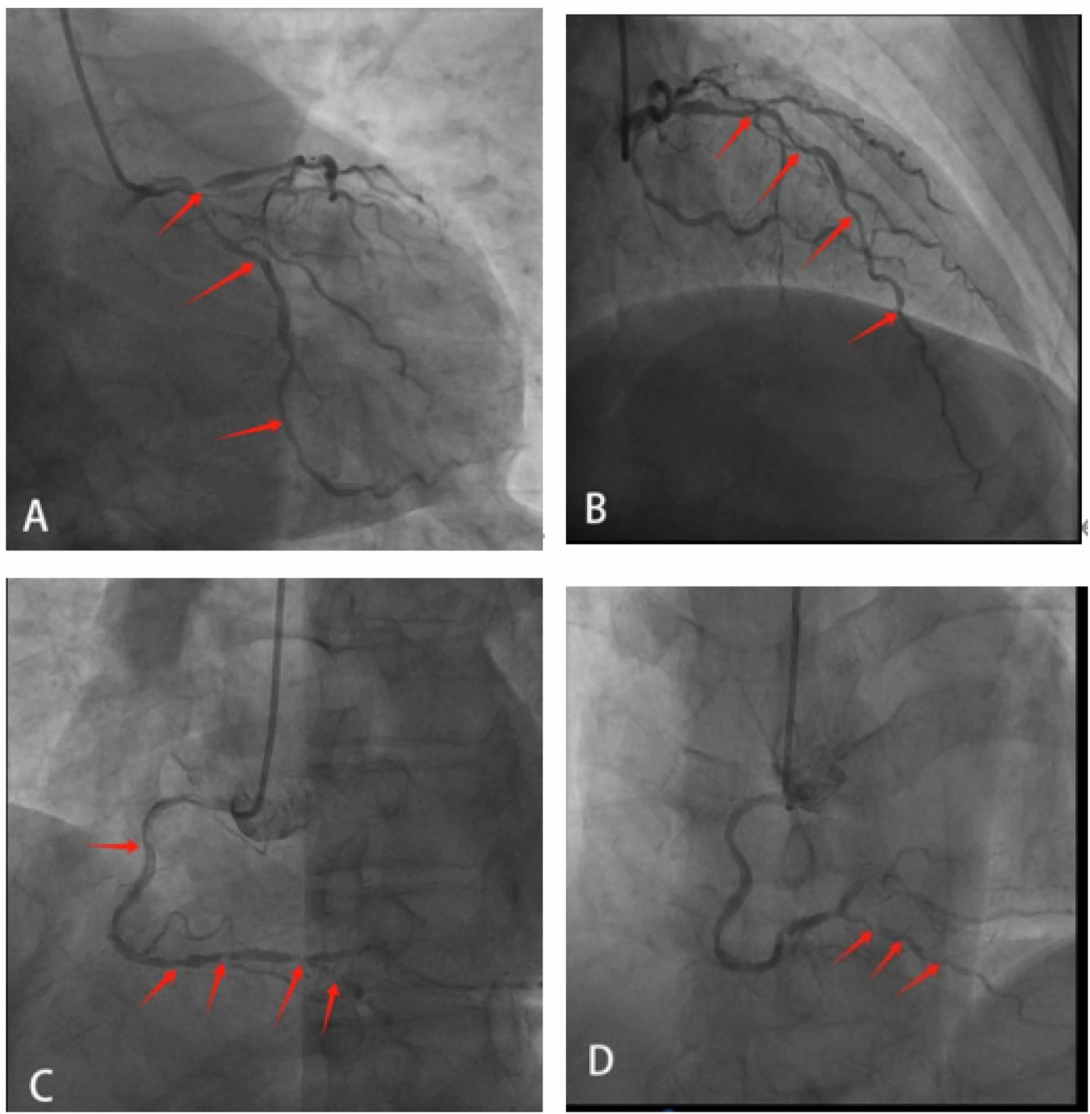
**(A)** LAD ostium stenosis approximately 95%. **(B)** Diffuse LAD mid-segment stenosis approximately 60%–85%. **(C)** RCA proximal mild-to-moderate stenosis, distal severe stenosis. **(D)** Diffuse PDA proximal-to-mid-segment stenosis.

**Figure 3:**
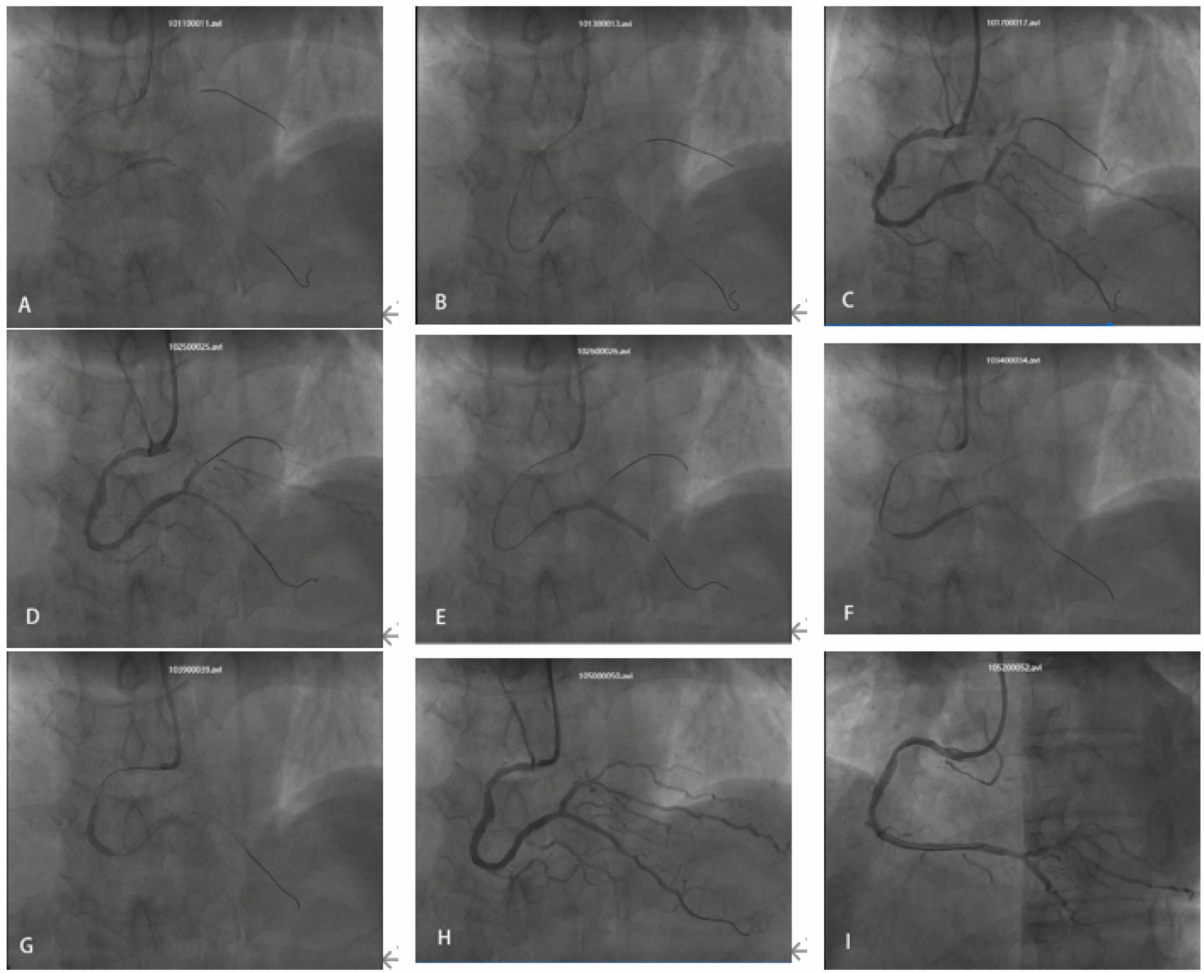
**(A)** Balloon angioplasty of RCA-PDA **(B)** balloon angioplasty of RCA mid-to-distal segment **(C,D)** balloon angioplasty of RCA mid-segment. **(E)** 2.5*33 mm stent **(F)** 2.75*33 mm stent **(G)** 3.0*23 mm stent **(H,I)** immediate postoperative outcome.

**Figure 4:**
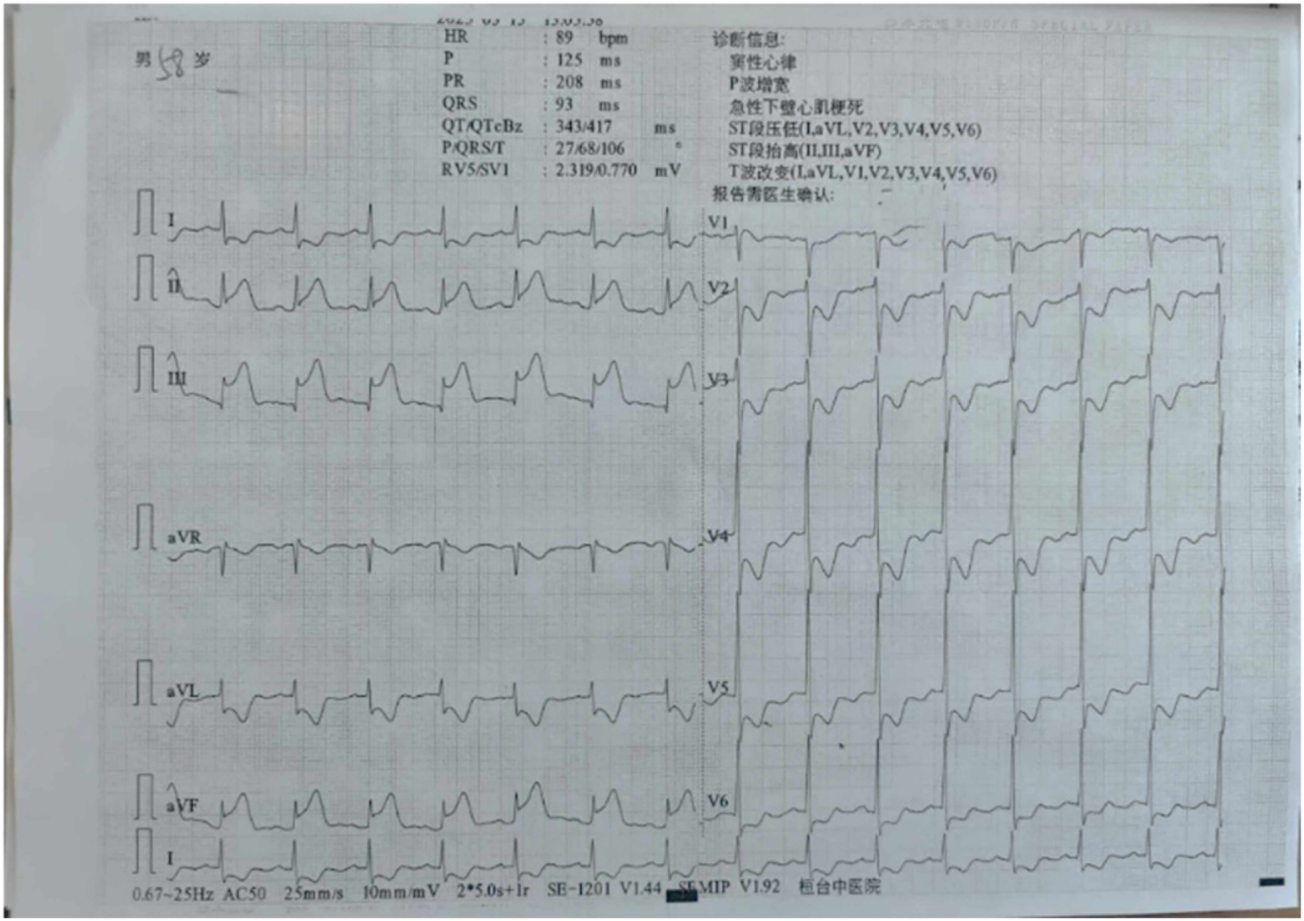
ST-segment elevation observed in leads II, III, and aVF.

**Figure 5:**
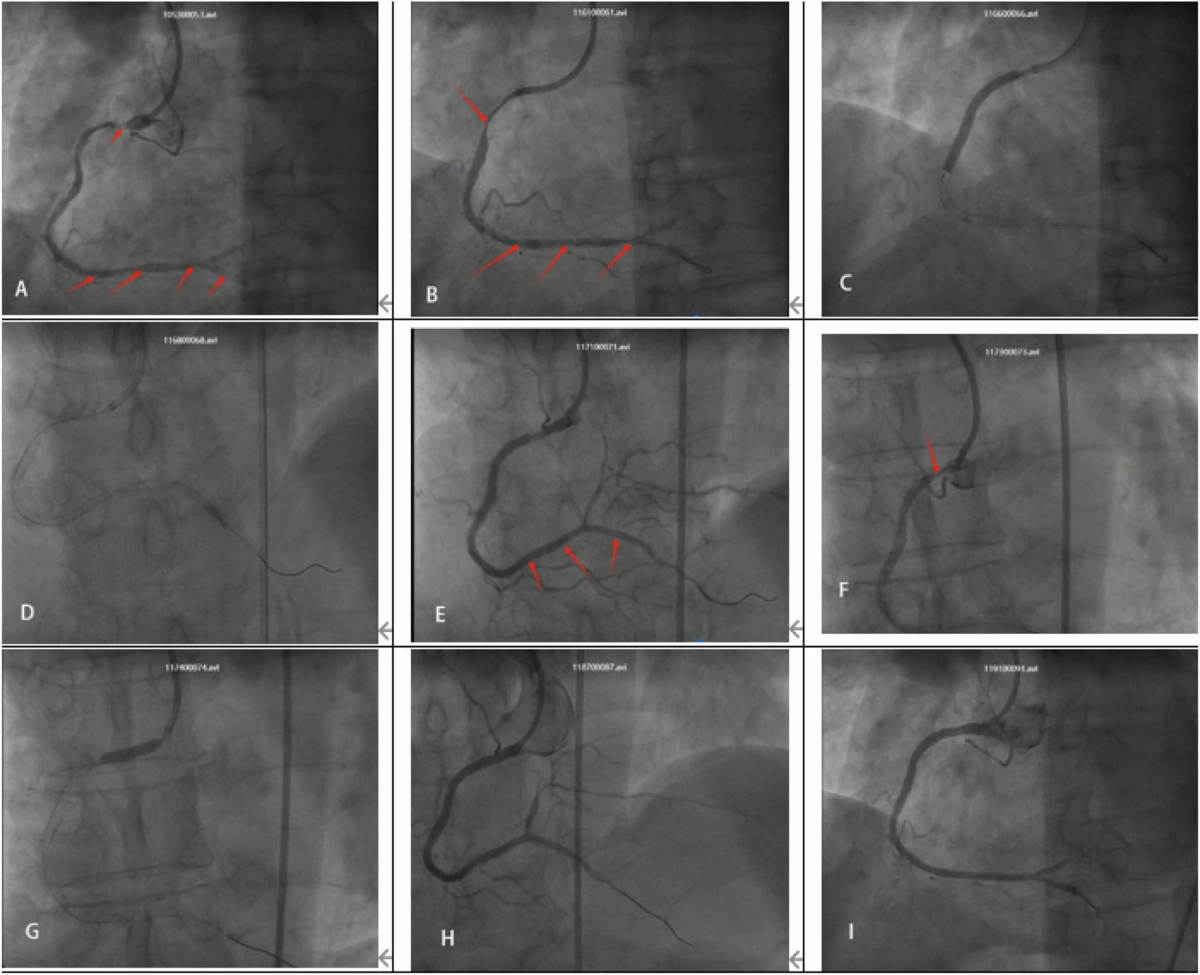
**(A)** Multiple thrombi in RCA proximal segment and within stent, red arrows. **(B)** Worsened mid-segment stenosis. **(C)** 3.5 × 33 mm stent **(D)** 1.5*15 mm Balloon angioplasty of distal PDA stent. **(E)** Thrombus in the mid-to-distal RCA stent disappeared after the intervention. **(F,G)** 3.5 × 13 mm stent implanted in RCA proximal segment. **(H,I)** The RCA stents were patent, with no evidence of thrombus formation, and TIMI flow grade 3 was achieved.

**Figure 6:**
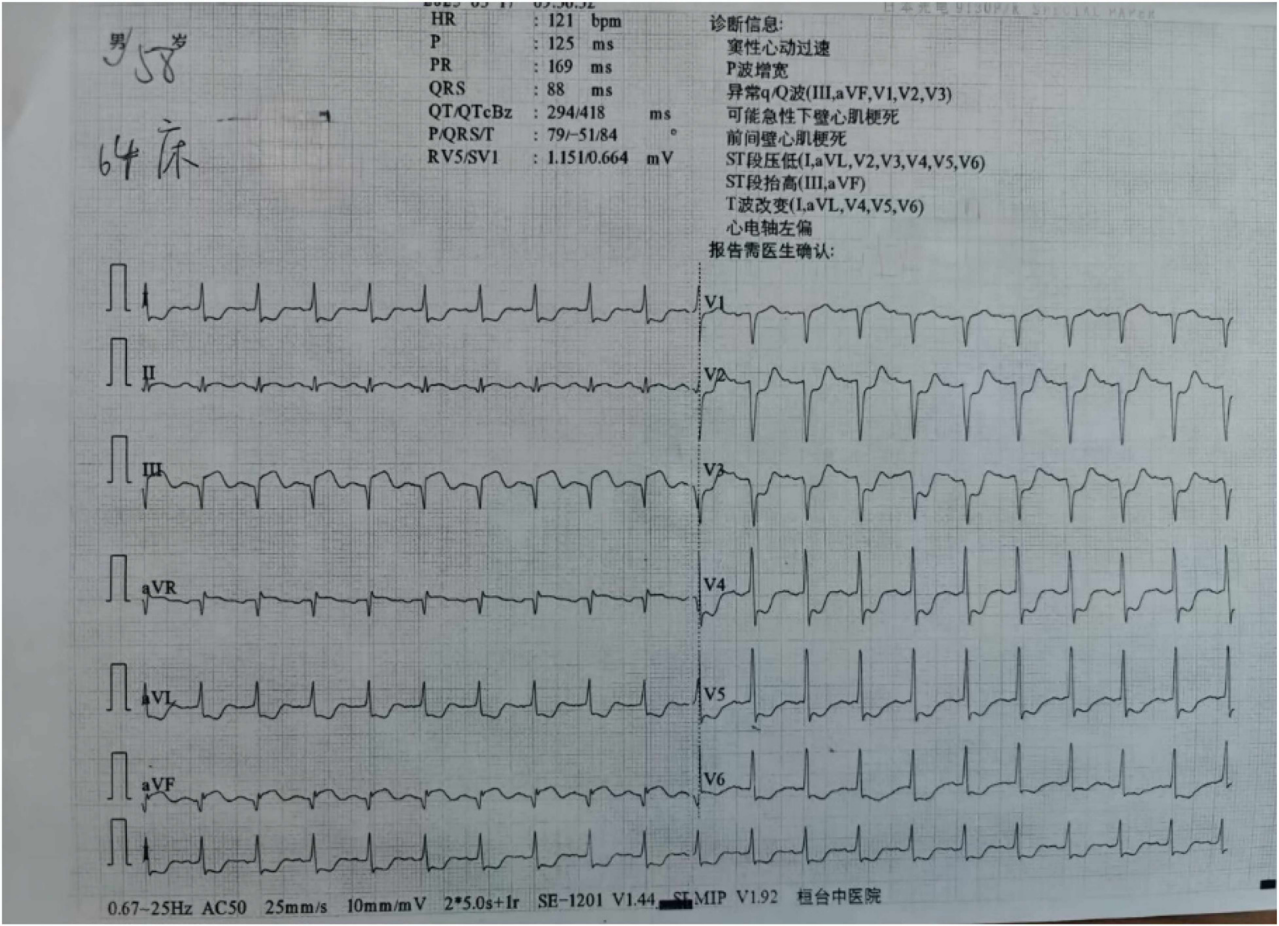
The ECG showed that the ST-segment in leads III and aVF had not fully normalized after PCI again.

On the second day of admission, the patient underwent coronary angiography after oral loading doses of aspirin and ticagrelor. The angiography revealed approximately 95% stenosis at the ostium of the left anterior descending artery (LAD), with diffuse stenosis of 60%–85% in the proximal to mid segments and TIMI grade 3 flow in the distal segment. The left circumflex artery (LCX) appeared hypoplastic with diffuse lesions, and the mid-segment exhibited 60%–80% stenosis. TIMI grade 3 flow was observed in the distal segment. The right coronary artery (RCA) showed approximately 70% stenosis in the proximal segment, 50% in the mid-segment, and diffuse stenosis ranging from 60%–99% distal to the second bend. The posterior descending artery (PDA) showed diffuse stenosis of 60%–80% in the proximal and mid segments, with TIMI grade 3 distal flow (Figure 2). Three Firebird2 stents (2.5 × 33 mm, 2.75 × 33 mm, and 3.0 × 23 mm) were implanted in the RCA (Figure 3). After the procedure was completed, tirofiban was initiated intravenously at a continuous infusion rate of 3 mL/h. Approximately 30 min after returning to the ward, the patient complained of recurrent chest tightness and discomfort. The repeated ECG exhibited notable ST-segment elevation in leads II, III, and aVF, accompanied by significant ST-segment depression in leads V2 to V5 (Figure 4). Acute inferior wall myocardial infarction was suspected and emergency coronary angiography via the femoral artery was promptly conducted by the catheterization laboratory. Repeated angiography revealed a substantial deterioration in RCA lesions, with approximately 90% stenosis in the proximal segment, 95% in the mid-segment, and focal in-stent thrombosis in the distal segment. Furthermore, the distal PDA demonstrated approximately 85% stenosis. Two additional stents were deployed in tandem from the ostium to the mid-segment of the RCA, while percutaneous transluminal balloon angioplasty was performed for the distal PDA lesion (Figure 5). Post-intervention, the patient's clinical symptoms gradually ameliorated. Considering the multiple tandem stent placements and suspected thrombus formation, the tirofiban infusion rate was increased to 5 mL/h. Four hours after the procedure, the patient's symptoms had markedly diminished, however, the follow-up ECG showed that the ST-segment in leads III and aVF had not fully normalized (Figure 6).

On postoperative day 2, laboratory tests showed significant elevations in cardiac biomarkers, including cardiac troponin I (cTnI) at >25 ng/mL, myoglobin (MYO) at 452 ng/mL, and N-terminal pro-brain natriuretic peptide (NT-proBNP) at 4,790 pg/mL. Afterwards, a critical alert was triggered by the complete blood count (CBC), indicating severe thrombocytopenia with a platelet count of 6 × 10^9^/L. Heparin-induced thrombocytopenia (HIT) was suspected. Subsequently, all antiplatelet and anticoagulant therapies, specifically tirofiban, aspirin, and ticagrelor, were promptly discontinued. Intravenous dexamethasone at a dosage of 10 mg was administered as an initial immunosuppressive measure. In the afternoon, a repeated CBC revealed a further decrease in platelet count to 2 × 10^9^/L. Peripheral blood smear revealed an increased proportion of white blood cells, rare scattered platelets, and elevated D-dimer level at 2.05 mg/L. Following this, the patient received intravenous methylprednisolone at a dosage of 40 mg and a subcutaneous injection of interleukin-11 (IL-11) at 1.5 mg.

In the morning of postoperative day 3, the platelet counts of the patients slightly increased to 7 × 10^9^/L. The peripheral smear showed leukocytosis with prominent granulation and enlargement in some neutrophils, basophilic stippled erythrocytes, and sparse, scattered platelets, including occasional giant platelets. The treatment regimen was maintained, including intravenous dexamethasone at 10 mg and subcutaneous IL-11 at 1.5 mg. Meanwhile, the patient developed symptoms of heart failure, which improved after the administration of appropriate heart failure medications. In the afternoon, the patient was transferred to a tertiary hospital with the requested of the patient's family. Upon arrival at the receiving hospital, re-evaluation revealed a critically low platelet count of 1 × 10^9^/L. The patient underwent treatment with intravenous immunoglobulin (IVIG) and platelet transfusions. On the second day post-transfer, approximately 30 min after the platelet transfusion, the patient experienced sudden onset of acute chest pain, followed by ventricular fibrillation, confirmed by continuous electrocardiographic monitoring. Despite immediate resuscitative measures, including defibrillation and cardiopulmonary resuscitation (CPR), the patient could not be revived and was pronounced dead.

**Figure 7:**
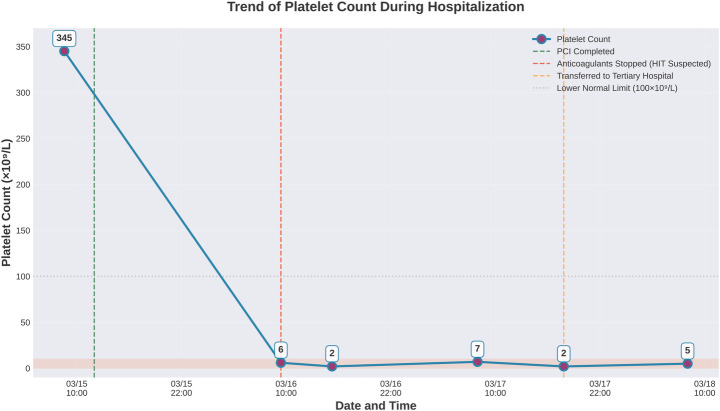
Platelet count trend chart.

**Figure 8:**
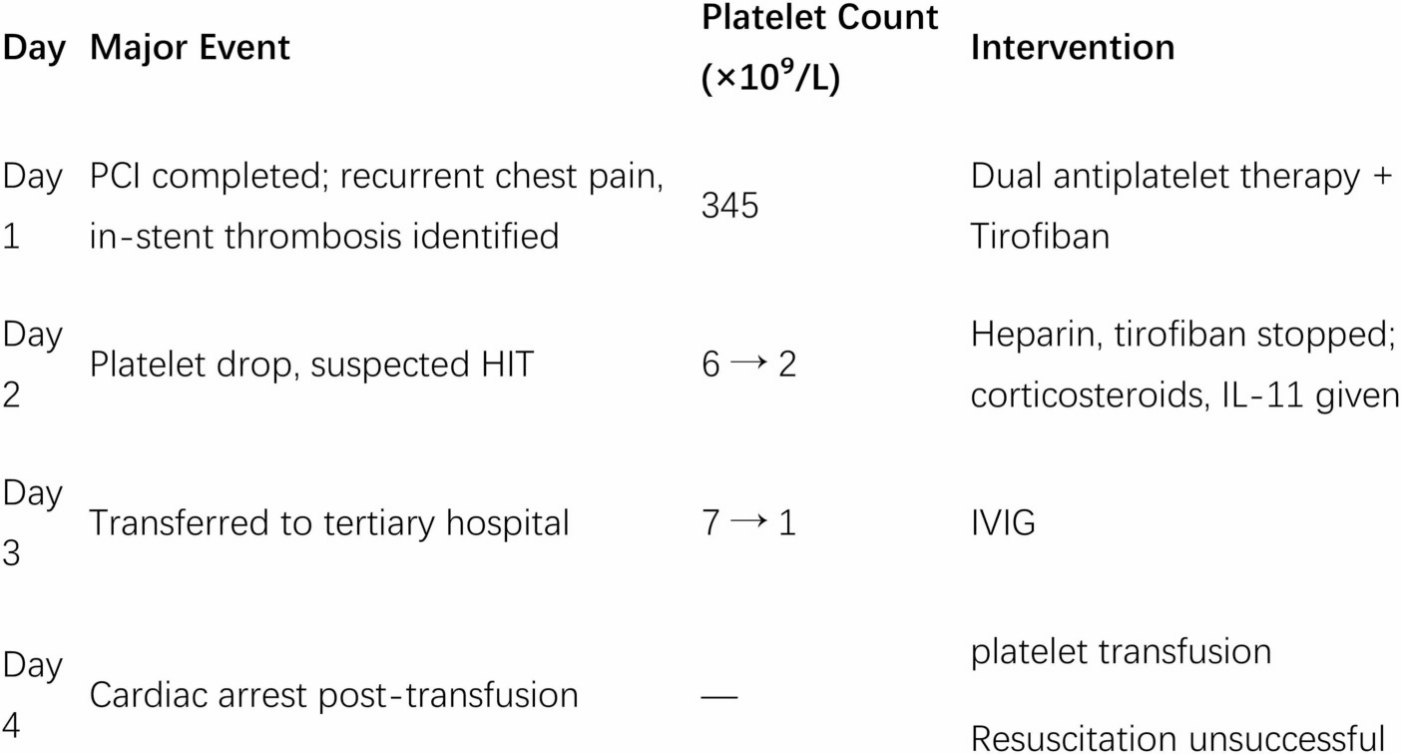
Graphical timeline illustrating major clinical events, interventions, and platelet count trends.

Timeline of major events before and after the patient's hospitalization, and the key indicators of platelet count are summarized in Table 1 and Figures 7, 8 as below.

**Table 1:** Timeline of major events before and after the patient's hospitalization.

One month ago	Chest tightness after the activity
Hospital day 1	
10:30	Blood tests and cardiac ultrasound were normal, but coronary angiography revealed triple vessel disease.
12:00	Three stents implanted in RCA, TIMI 3 flow achieved, no patient discomfort during procedure.
12:30	Thirty minutes post-procedure, patient experienced recurrent chest discomfort; ECG showed ST-segment elevation in leads II, III, and aVF.
12:45	Repeat angiography showed worsening RCA lesions and thrombus formation in stents
13:30	Two additional stents placed, chest pain improved, and TIMI flow 3 restored.
16:30	Chest pain resolved, ST-segment returned to normal
Hospital day 2	
09:24	Platelet count decreased to 6 × 10^−9^/L, dual antiplatelet therapy and tirofiban discontinued; Dexamethasone 10 mg given.
15:16	Platelet count dropped further to 2 × 10^−9^/L, Methylprednisolone 80 mg administered
Hospital day 3	
07:57	Platelets improved to 7 × 10^−9^/L, but heart failure developed; condition improved after treatment and transfer.
17:50	Platelet count dropped to 2 × 10^−9^/L.
Hospital day 4	
08:02	Platelet count 5 × 10^−9^/L, one therapeutic dose of platelet transfusion
10:30	Recurrent chest pain and ventricular fibrillation, patient passed away.

## Discussion

The diagnosis of HIT post-PCI presents significant challenges due to its clinical overlap with other post-procedural complications, such as in-stent thrombosis, restenosis, and device-related vascular injury (3). In this case, the patient experienced an acute myocardial infarction (AMI) shortly after PCI, which was initially attributed to potential stent-related issues. However, the rapid onset of severe thrombocytopenia necessitated the consideration of HIT. Due to the lack of confirmatory anti-PF4/heparin antibody testing at our institution, the diagnosis remained clinically suspected based on the 4Ts scoring system and clinical presentation (4–6), rather than laboratory-confirmed. A major limitation of this case was the lack of confirmatory anti-PF4/heparin antibody testing (ELISA or functional assays such as SRA/HIPA), which were unavailable at our institution. Consequently, the diagnosis relied on clinical features and the 4Ts score. This diagnostic gap is acknowledged as a limitation that may introduce uncertainty in confirming HIT. This case underscores the importance of maintaining a high index of suspicion for HIT in patients presenting with unexpected thrombocytopenia and thrombotic events post-PCI.

A critical diagnostic consideration in this case was whether the thrombocytopenia could be attributed to tirofiban, a glycoprotein IIb/IIIa inhibitor commonly used during PCI. Tirofiban-induced thrombocytopenia (TIT) is characterized by acute and severe reductions in platelet counts (7), typically occurring within 1 to 24 h after drug initiation, with the most rapid declines observed within minutes of infusion. A hallmark feature of TIT is bleeding, and platelet recovery is usually rapid, with counts normalizing within 2 to 5 days after discontinuation of the drug due to tirofiban's short half-life. In contrast, this patient exhibited no bleeding manifestations, and platelet counts did not significantly improve following the cessation of tirofiban, rendering TIT an unlikely diagnosis (8). The concurrent presence of thrombosis and persistent thrombocytopenia further supports HIT as the primary etiology.

HIT is characterized by a paradoxical coexistence of thrombocytopenia and a prothrombotic state, driven by immune-mediated platelet activation (9). The formation of anti-PF4/heparin antibodies leads to platelet aggregation and microthrombus formation, thereby increasing the risk of arterial and venous thromboembolism (10). HIT is classified into two types: Type I, a non-immune-mediated and benign condition associated with mild, early thrombocytopenia (occurring within 5 days of heparin exposure), and Type II, an immune-mediated process that encompasses multiple subtypes, including typical-onset HIT (5–10 days post-heparin), rapid-onset HIT (within 24 h, typically following prior heparin exposure), delayed-onset HIT (up to 3 weeks post-cessation), and spontaneous HIT (occurring without prior heparin exposure, often linked to recent infection or surgery). In this case, given the absence of prior heparin exposure and the rapid onset of severe thrombocytopenia and thrombosis, this presentation is most consistent with clinically suspected autoimmune HIT with rapid-onset features, though the absence of confirmatory testing precludes definitive classification (11).

The management of HIT in the setting of PCI necessitates the immediate cessation of heparin and the initiation of non-heparin anticoagulants, such as argatroban or bivalirudin, which directly inhibit thrombin without cross-reactivity with PF4/heparin antibodies (12). In this case, the transition to alternative anticoagulation was critical but complicated by the patient's recurrent thrombotic events. The use of immunosuppressive therapy, such as methylprednisolone, aimed to mitigate the immune-mediated component of HIT, but yielded limited benefit (13), as evidenced by the patient's persistent thrombocytopenia and eventual demise. This outcome underscores the unpredictable and often fatal nature of HIT, particularly in patients with multi-vessel coronary artery disease, where the prothrombotic environment exacerbates the risk of adverse events (14). Furthermore, the administration of platelet transfusions, as occurred in this case, is generally contraindicated in HIT due to the risk of worsening thrombosis, highlighting the importance of adherence to evidence-based guidelines (15). In this case, corticosteroids, IL-11, and platelet transfusions were administered empirically to counter severe thrombocytopenia before confirmatory testing was available. However, these measures are not recommended in HIT management. Guideline-endorsed therapy with non-heparin anticoagulants, such as argatroban or bivalirudin, was considered but could not be initiated due to restricted access and rapid clinical decline. This highlights a real-world limitation in resource-constrained settings.

A critical aspect of this case requiring explicit acknowledgment is the potential iatrogenic contribution to the fatal outcome. Following transfer to the receiving hospital, the patient received platelet transfusion, after which—approximately 30 min later—he developed sudden acute chest pain and ventricular fibrillation leading to death. While direct causality cannot be definitively established, platelet transfusion in HIT is strongly contraindicated as it may paradoxically fuel the prothrombotic state by providing additional substrate for anti-PF4/heparin antibody-mediated platelet activation and subsequent thrombosis. This temporal association raises significant concern that the platelet transfusion may have contributed to acute stent thrombosis and the fatal arrhythmic event. This case underscores that inadequate recognition of suspected HIT during inter-hospital transfer can lead to well-intentioned but potentially harmful interventions, highlighting the critical importance of clear communication of suspected diagnoses during patient handoffs.

This case serves as a stark reminder of the lethal potential of HIT in the PCI population. Routine monitoring of platelet counts is essential for the early detection of HIT, with a decline of ≥50% from baseline or an absolute count of <100,000/µL necessitating immediate evaluation for HIT (16). The integration of rapid diagnostic assays, such as enzyme-linked immunosorbent assays for anti-PF4/heparin antibodies, could expedite diagnosis and improve patient outcomes (17). Furthermore, multidisciplinary collaboration among interventional cardiologists, hematologists, and pharmacists is critical for optimizing anticoagulation strategies and mitigating thrombotic risk. Standardized protocols for post-PCI platelet monitoring and the early consideration of HIT in patients presenting with recurrent ischemia or electrocardiographic changes are essential to prevent delays in diagnosis and treatment (18).

In this case report, several limitations must be acknowledged. For example, the reliance on clinical and serological criteria for diagnosing HIT introduces potential diagnostic uncertainty, particularly in the absence of real-time functional assays, such as the serotonin release assay (19). The complexity of the patient's coronary anatomy and their rapid clinical deterioration may limit the generalizability of this case to less severe presentations. Furthermore, current management deviations (e.g., corticosteroids, platelet transfusions) highlight the need for updated protocols (20). Future focus should include rapid HIT diagnostics, novel HIT-specific anticoagulants, spontaneous HIT prevention strategies, enhanced clinician education on atypical presentations, and standardized post-PCI monitoring. These measures are essential for reducing morbidity and mortality associated with HIT (21).

An essential learning point from this case is the extreme risk posed by complete withdrawal of antithrombotic therapy in a patient with extensive multivessel stenting (five stents total) and active thrombosis. The cessation of all antiplatelet and anticoagulant agents—though necessary given the suspected HIT—created a critical therapeutic vacuum in a patient at extraordinarily high risk for stent thrombosis. This risk was further compounded by the patient's transfer to another hospital, where treatment continuity was disrupted and awareness of the clinical situation may have been incomplete. In ideal circumstances, immediate transition to a non-heparin anticoagulant (such as argatroban or bivalirudin) should occur simultaneously with heparin cessation. The absence of these agents, combined with the transfer-related care fragmentation, left the patient without any antithrombotic protection during a period of maximum vulnerability. This underscores the imperative for healthcare systems to ensure: (1) ready availability of alternative anticoagulants, (2) seamless communication during inter-hospital transfers, and (3) clear protocols for managing suspected HIT in patients with recent coronary intervention.

## Conclusion

In conclusion, this case illustrates the diagnostic and therapeutic challenges of clinically suspected HIT in patients undergoing PCI. In the absence of confirmatory anti-PF4/heparin antibody testing, the diagnosis relied on clinical criteria and the 4Ts scoring system, highlighting the need for wider availability of rapid diagnostic assays (22). This case underscores several critical lessons: (1) the paramount importance of maintaining a high index of suspicion for HIT in patients presenting with unexpected thrombocytopenia and thrombotic events post-PCI; (2) the absolute necessity of guideline-recommended non-heparin anticoagulation (argatroban or bivalirudin) as first-line therapy when HIT is suspected, rather than empiric measures such as corticosteroids or platelet transfusions which may be ineffective or harmful (23, 24); (3) the extreme danger of complete antithrombotic withdrawal in patients with extensive coronary stenting without immediate transition to alternative anticoagulation; and (4) the critical importance of system-level preparedness, including ready access to alternative anticoagulants and clear protocols for suspected HIT management, particularly during inter-hospital transfers where care fragmentation can lead to adverse outcomes.

The fatal outcome in this case—temporally associated with platelet transfusion administered at the receiving hospital following transfer—serves as a sobering reminder that inadequate recognition of suspected HIT can lead to contraindicated interventions with potentially catastrophic consequences. This case also contributes to the growing recognition of autoimmune HIT as a distinct entity requiring increased vigilance for atypical presentations in PCI patients (25). Future efforts should prioritize: development and implementation of rapid bedside HIT diagnostics, ensuring universal availability of non-heparin anticoagulants, establishing standardized HIT management protocols across healthcare systems, enhancing clinician education on both typical and atypical HIT presentations, and improving inter-hospital communication to ensure continuity of care. These system-level improvements are essential to prevent similar tragic outcomes in this high-risk patient population.

## Patient perspective

The patient's family expressed shock and grief over the rapid deterioration following PCI. They emphasized the importance of clear communication regarding rare but severe complications like HIT and expressed their willingness to share this case to raise awareness among clinicians and patients alike.

## Data Availability

The raw data supporting the conclusions of this article will be made available by the authors, without undue reservation.
